# The Heritability of Type D Personality by an Extended Twin-Pedigree Analysis in the Netherlands Twin Register

**DOI:** 10.1007/s10519-020-10023-x

**Published:** 2020-10-16

**Authors:** Ruifang Li-Gao, Dorret I. Boomsma, Eco J. C. de Geus, Johan Denollet, Nina Kupper

**Affiliations:** 1 CoRPS Center of Research On Psychology in Somatic Diseases, Tilburg University, Tilburg, The Netherlands; 2 Department of Biological Psychology, Vrije Universiteit, Amsterdam, The Netherlands; 3 Dept of Medical and Clinical Psychology, Center of Research On Psychology in Somatic Diseases (CoRPS), Tilburg University, P. O. Box 90153, 5000 LE Tilburg, The Netherlands

**Keywords:** Pedigree analysis, Heritability, Type D personality, Negative affectivity, Social inhibition, Genetic non-additivity, Shared household effect, Extended twin-family design, Netherlands Twin Register

## Abstract

Type D (*Distressed)* personality combines negative affectivity (NA) and social inhibition (SI) and is associated with an increased risk of cardiovascular disease. We aimed to (1) validate a new proxy based on the Achenbach System of Empirically Based Assessment (ASEBA) for Type D personality and its NA and SI subcomponents and (2) estimate the heritability of the Type D proxy in an extended twin-pedigree design in the Netherlands Twin Register (NTR). Proxies for the dichotomous Type D classification, and continuous NA, SI, and NAxSI (the continuous measure of Type D) scales were created based on 12 ASEBA items for 30,433 NTR participants (16,449 twins and 13,984 relatives from 11,106 pedigrees) and sources of variation were analyzed in the ‘Mendel’ software package. We estimated additive and non-additive genetic variance components, shared household and unique environmental variance components and ran bivariate models to estimate the genetic and non-genetic covariance between NA and SI. The Type D proxy showed good reliability and construct validity. The best fitting genetic model included additive and non-additive genetic effects with broad-sense heritabilities for NA, SI and NAxSI estimated at 49%, 50% and 49%, respectively. Household effects showed small contributions (4–9%) to the total phenotypic variation. The genetic correlation between NA and SI was .66 (reflecting both additive and non-additive genetic components). Thus, Type D personality and its NA and SI subcomponents are heritable, with a shared genetic basis for the two subcomponents.

**Electronic supplementary material:**

The online version of this article (10.1007/s10519-020-10023-x) contains supplementary material, which is available to authorized users.

## Introduction

Type D or *Distressed* personality is an established risk factor for the development and poor prognosis of coronary artery disease (Beutel et al. 2012; Denollet et al. 2013; Grande et al. 2012; Kupper and Denollet 2018; Wang et al. 2016). Type D personality also has been identified as a vulnerability factor for mental disorders such as anxiety and depression in cardiac patients (Kupper et al. 2013; Martens et al. 2008) and in the general population (Kupper and Denollet 2014; Svansdottir et al. 2012; van Dooren et al. 2016). Type D personality is characterized by a combination of two traits: social inhibition (SI; i.e., the tendency not to share and express emotions in social interactions, because of fear of rejection or disapproval from others) and negative affectivity (NA; referring to the tendency to feel negative emotions (e.g., anger, sadness, fear, and irritability) across time and situations. A cut-off of 10 for both continuous subcomponent scores shows the prevalence of Type D to be about one in four in patients with heart disease, compared to one in five in the general population (Denollet 2000). Type D personality can also be described as a continuous trait, using the multiplicative interaction of SI by NA. Multiple biological (e.g., systemic inflammation, endothelial dysfunction, autonomic nervous dysregulation, telomere shortening) and behavioural (e.g., sleep problems, poor social support, poorer self-management skills) pathways are associated with Type D personality and may explain its adverse effects (Denollet et al. 2018; Jandackova et al. 2017; Kupper and Denollet 2018; Schoormans et al. 2018).

Type D personality is typically assessed by the 14-item Type D Scale (DS14) (Denollet 2005), which combines two subscales: the NA (Negative Affectivity) subscale gauging the tendency to experience negative emotions and the SI (Social Inhibition) subscale, which reflects the tendency to inhibit the expression of negative emotions in social interaction. Existing cohorts enriched with biological and genetic data often lack Type D personality measured by DS14, which greatly hinders large-scale studies of Type D personality and its pathophysiological effects leading to diseases. From classical twin design studies, the dichotomous Type D personality classification, as well as its two underlying continuous subcomponents (NA and SI) were shown to be heritable (52%, 46% and 50% respectively) and stable over time (Kupper et al. 2011, 2007). The classical twin design allows estimation of genetic and non-genetic variance components, but does not allow simultaneous estimation of genetic non-additive (dominance) variance (D) components together with variance components linked to shared household or other common (C) environment sharing (Boomsma et al. 2018; Keller et al. 2010; Posthuma and Boomsma 2000; Rebollo and Boomsma 2006). However, many twin registries nowadays have recruited not only twins, but also their family members, including parents, siblings, spouses and offspring of twins, which allows for the construction of extended pedigrees, including biological and non-biological family members. Such extended twin family designs increase the estimation accuracy and robustness to violations of assumptions for the classical twin models, and allow for simultaneous estimation of non-additive and shared environmental variance components (Keller et al. 2010). In the current study, we employ an extended twin-pedigree design to analyze individual differences in Type D personality. Our main goals were two-fold. We first aimed to validate a new proxy for Type D and its SI and NA subscales from items of the Adult Self-Report (ASR) of the Achenbach System of Empirically Based Assessment (ASEBA) in two samples (young adults and general population) who completed candidate ASEBA items, the DS14, and several other personality and mood questionnaires. Second, we aimed to estimate the heritability of these Type D proxies by an extended twin-pedigree analysis in the Netherlands Twin Register (NTR).

## Methods

### Participants

Adult participants in the NTR completed longitudinal surveys on health, lifestyle and personality from 1991 onward, with the study population comprising (young) adult twins, and their biological and non-biological family members. All procedures performed in studies involving human participants were in accordance with the ethical standards of the institutional and/or national research committee and with the 1964 Helsinki declaration and its later amendments or comparable ethical standards. The studies were approved by the Central Ethics Committee on Research Involving Human Subjects of the VU University Medical Center, Amsterdam, an Institutional Review Board certified by the U.S. Office of Human Research Protections (IRB Number IRB-2991 under Federal-wide Assurance-3703; IRB/Institute Codes, NTR 03-180). The details on the recruitment procedure and sample characteristics have been detailed elsewhere (Boomsma et al. 2006, 2002). Relationships among NTR participants is based on information from surveys, NTR non-survey studies and the Person Administration of the Netherlands Twin Register (PANTER) database, which is a person-oriented database that specifies twin-twin, sibling-sibling, parent-offspring, spouse-spouse and teacher–pupil relations (the latter we do not consider here). In the current version of the PANTER database 255,785 NTR participants are included with information on these relationships. An algorithm described as previously (Boomsma et al. 2018) was developed to create pedigrees of nuclear families or more extended family trees. The types of relatives considered in the pedigree included *MZ twin pairs* (additive sharing = 100%, dominance sharing = 100%), *DZ twin pairs* (additive sharing = 50%, dominance sharing = 25%), *non-twin siblings*: sib-sib pairs and twin-sib pairs (additive sharing = 50%, dominance sharing = 25%), *mother/father/daughter/son* (additive sharing = 50%, dominance sharing = 0%), *grandmother/grandfather/granddaughter/grandson* (additive sharing = 25%, dominance sharing = 0%), *avuncular* (i.e. the genetic relationship between aunts and uncles and their nieces and nephews with additive sharing = 25%, dominance sharing = 0%), *first cousin* (i.e. the people who have two of the same grandparents with additive sharing = 12.5%, dominance sharing = 0%), *first cousin with MZ twins as parents* (additive sharing = 25%, dominance sharing = 0%), *double first cousin* (additive sharing = 25%, dominance sharing = 6.25%) and *spouse* (additive sharing = 0%, dominance sharing = 0%). Zygosity information for twins and multiples was obtained from blood group or DNA markers, and survey data, to indicate the co-twins as either monozygotic (MZ) or dizygotic (DZ) twins (Ligthart et al. 2019). Within the entire NTR database, there were 31,116 participants with ASEBA data from at least one survey (Supplemental Fig. 1).

### Validation of the Type D proxy by ASEBA

We inspected items from Adult Self-Report (ASR) of the Achenbach System of Empirically Based Assessment (ASEBA) (Achenbach et al. 2017) for their similarities with respect to content and wording with the original DS14 questionnaire (Denollet 2005). Twelve (six NA and six SI items) items were shortlisted to derive the Type D personality proxy scale. For the SI subcomponent, the following six items were adopted: (1) ASEBA75-Too shy or timid; (2) ASEBA67-Trouble making and keeping friends; (3) ASEBA69-Reserved, keep things to self; (4) ASEBA42-Rather be alone than with others; (5) ASEBA111-Keep from getting involved with others; (6) ASEBA48-Not liked by others. For the NA subcomponent, another six items from ASEBA were used: (1) ASEBA103-I am unhappy, sad, or depressed; (2) ASEBA112-I worry a lot; (3) ASEBA50-I am too fearful or anxious; (4) ASEBA45-I am nervous or tense; (5) ASEBA115-I feel restless; (6) ASEBA87-Moods and feelings change suddenly. Each item from the ASEBA was rated on a 3-point Likert scale from 0 (never), 1(sometimes) to 2 (often), and total scores for NA and SI thus could range from 0 to 12.

In the original DS14 questionnaire, the two Type D subcomponents are assessed by seven items with a 5-point Likert scale ranging from 0 = false to 4 = true. The range of each subscale is between 0 and 28, and a score of 10 or higher on both subscales in the DS14 classifies a person as having a Type D personality. More recently, studies have suggested the use of a continuous measure of Type D personality, more reflective of the continuity of the underlying dimensions (Ferguson et al. 2009), which is operationalized as the multiplicative interaction of NAxSI. For historical reasons, the dichotomy of Type D was still considered in the validation analysis.

#### Validation samples

The original DS14 questionnaire and the 12 candidate ASEBA items were administered simultaneously in two independent samples. The first is a sample of undergraduate students from Tilburg University (N = 214, 91% women, 87% European descent), while the second was a general population sample of 680 people (55% women, average age = 49 ± 16 years). Detailed sample descriptions are provided in the online Supplement.

### Assessment of Type D personality and its subcomponents in NTR

After validation, 12 ASEBA items were retained to assess social inhibition and negative affectivity in the NTR dataset. We calculated sum scores for NA and SI. If two or less items were missing, values were replaced by the person mean of the other available subscale items. Validation concluded that a cut-off of 3 on the ASEBA NA and SI scales was optimal to derive the dichotomous Type D classification (Online supplement). Since there has been a discussion whether Type D is better represented by the interaction of the two underlying traits (i.e. NAxSI), instead of the dichotomy based on the cut-offs of both underlying traits (Ferguson et al. 2009; Lodder 2020), we defined the interaction (NAxSI) of the continuous subscale total scores as a continuous measure of Type D. For historical reasons, the dichotomy of Type D was considered in the descriptive results, but no variance decomposition analyses were carried out.

### Statistical analysis

#### Proxy validation

To examine whether the ASEBA-based proxy measures captured the same constructs as the DS14, we performed three series of analyses. First, we used the student sample to examine initial validity, i.e. exploratory factor analysis and reliability analysis with item-total statistics, and we determined a potential cut-off. Then, in the second sample, which had an equal sex and age distribution, we performed reliability analysis, confirmatory factor analysis and an analysis of measurement invariance with respect to sex and age categories. Thirdly, we examined the ‘diagnostic accuracy’ of the proxy cut-off we identified against the gold standard (i.e. DS14) by receiver operating characteristic (ROC) curves. We repeated the confirmatory factor analysis (CFA) and measurement invariance analysis in a subset of the unrelated NTR participants, i.e. all dyads of parents of twins. For details on these analyses of the ASEBA-based proxy measures, we refer to the online supplement.

### Extended twin-pedigree analysis in the NTR

#### Survey selection and imputation

The longitudinal ASEBA questions that are relevant for the Type D proxy scale calculation were selected from the first (1991, wave-1) and follow-up surveys in 1995 (wave-3), 1997 (wave-4), 2000 (wave-5), 2009 (wave-8) and 2013 (wave-10) of NTR data collection in late-adolescents and adults. First, the survey with the most complete answers on all twelve proxy items was selected for all individuals. When there were more surveys with complete data for an individual, the survey with most other relatives from the individual’s pedigree was selected (Supplemental Fig. 1). This led to a total of 28,709 individuals with complete answers in at least one survey, and 2407 individuals without complete answers in any survey; this is largely explained by changes in the content of the ASEBA instrument for Young and Adult Self Report over the past 30 years. For these 2407 individuals, the survey with the largest number of completed items was chosen, and up to two missing items for the 6-item NA and SI proxy scales were imputed from previous surveys of that same individual having answers on the items. If these were not available, the missing items were imputed as the mean of NA and SI sum scores. If a person had three or more missing answers in all waves for either NA or SI, the entire subscale was set to missing. The dichotomous Type D proxy was based on the joint cut-off scores for NA and SI determined during the validation procedure (see online supplement for derivation of cut-off). If a score was missing for either NA or SI while the other scale was present and lower than the pre-defined proxy cut-off, the individuals were automatically classified as non-Type D personality, as a value above the cut-off on both subscales is needed to classify as having a Type D personality. After this two-step imputation, there were 30,433 NTR participants (40% male) with valid Type D personality assessments by the proxy scale, with nearly similar numbers for NA and SI subscales (N = 30,857 and N = 29,685 respectively). The average age at which Type D personality was determined was 36.6 (SD = 16.2) years (the average age in males/females: 37.1/36.2 years; the average age of parents 55.8 years, of twins 28.3 years).

#### Pedigree input file for Mendel

Twins and multiples accounted for 54% of the participants and most twins came from complete pairs (80% of the sample). The numbers of complete pairs of MZ and DZ twins were 3187/3382 for NA subscale, 3093/3233 for SI subscale, 3144/3322 for dichotomous Type D measures. Family members were defined as sharing a household if they were spouses, parents and their offspring not older than 18 (Boomsma et al. 2018). The ‘Mendel’ software transitioned from linkage analysis software to a more comprehensive package that can model the covariance matrix of family members based on the known genetic relations of family members and the specification of household sharing. Genetic correlations among family members for the genotype (additive and non-additive) values follow from the biometrical model and are derived by the software from the pedigree structure. Pedigrees were defined based on the entire NTR databases to optimize information on relations among participants and merged to the phenotype information on the 30,433 participants with Type D data.

### Heritability of Type D personality and its subcomponents assessed by the proxy scale

To obtain a general idea of the familial resemblance of Type D personality measures, correlations were estimated for MZ/DZ twins and siblings, stratified by sex to examine possible sex effect modification in Type D personality (Kupper et al. 2007). For this same purpose, correlations were calculated separately for father-son, father-daughter, mother-son and mother-daughter pairs. Lastly, correlations between spouses, mostly fathers and mothers of twins, but also correlations between twin-spouse pairs were compared. These spouse correlations give information on whether a longer period of household sharing will increase the similarity on Type D personality, as father and mothers of twins share a household for longer than twins and their spouses (21). For the dichotomous trait (i.e. Type D classification, based on the diagnostic cut-off), tetrachoric correlations were derived by the R function “polychor” in the package “polycor” (Fox 2019). For the continuous measures (i.e. NA, SI and NAxSI), Pearson correlations were derived by the R base function “cor”. To estimate the 95% confidence interval (95% CI) for correlations, bootstrapping was applied as implemented in the R function “cor.ci” in the package “psych” (Revelle 2019).

To estimate heritability, the proportion of phenotypic variation accounted for by additive genetic factors (A) was estimated, together with the proportion of variation accounted for non-additive, or dominance (D), genetic factors (these two sum to the broad-sense heritability). In addition, the model included variance components for shared household effects (H) and unique environment (E). Genetic univariate analyses were carried out for the ASEBA-proxy for the continuous NA and SI subscales, and for the continuous NAxSI trait (i.e. continuous Type D).

We first ran a series of univariate analyses on each of the 3 continuous measures in ‘Mendel’ (Lange et al. 2013), with age and sex included in all the analyses as fixed effects. Inverse normal transformation was applied to the continuous measures (i.e., NA, SI and NAxSI) to eliminate right skewness (Supplemental Fig. 2). For comparison, we gauged the effect of the inverse normal transformation, by also calculating heritability estimates based on the untransformed scores, with results showing that heritability estimates were very comparable (Supplemental Table 1).

Nested sub-models were considered in the analyses, and a likelihood ratio test was applied to indicate the relative fit of a reduced model (either ADE, AHE or AE) compared with the full model with components A, D, H and E (Dominicus et al. 2006).

### Bivariate analysis of NA and SI

To investigate whether the NA and SI subscales were influenced by a common set of genetic or environmental factors, bivariate genetic analyses were performed between NA and SI in ‘Mendel’. The full model was defined as the model with ADHE factors. A number of nested models were compared to the full model and tested by likelihood ratio tests, with age and sex as fixed effects in all models. The genetic and environmental correlations were obtained from standardizing the A, D, H, and E covariance matrices. We also obtained the proportion of phenotypic covariance between NA and SI explained by genetic and non-genetic factors.

## Results

### Summary of validation results

A detailed description of the proxy validation results can be found in the online Supplement. In summary, reliability analysis in multiple validation samples showed that the ASEBA derived scales were internally consistent. Exploratory factor analysis showed the presence of two factors (NA and SI) that held up in the confirmatory factor analyses in two different samples. Moreover, when adding DS14 and ASEBA items to a two-factor model, this did not produce a worse fit compared to the 4-factor model in which DS14 and ASEBA factors were defined separately. Measurement invariance analysis with respect to age categories and sex showed that in the general population sample, there was equality between age categories in the factor configuration, item loadings, and thresholds. In the general population, the item means differed for the subsequent age categories. For sex, similar findings were obtained. Within the NTR parent dyad subset, there was measurement equivalence for age categories at all examined levels. With respect to sex, analysis concurred with the results of the general population sample, showing sex differences for item means.

Construct validity analysis in the general population sample showed that the ASEBA derived scales correlated in a similar degree and direction as DS14 derived scales to personality and mood constructs that were thought of as concordant (e.g., neuroticism and NA) and discordant (e.g., extraversion and SI). Intra-class-correlations showed that agreement between measures was good (*r* > 0.65), certainly when considering the difference in item range (3 vs 5-point Likert scale). Prevalence of Type D personality was 21% according to the ASEBA based proxy and 23% according to the gold standard DS14. ROC analysis determined that with the cut-off of three, the ASEBA derived NA and SI scales predicted the gold standard Type D classification very well, with good diagnostic accuracy. In sum, we conclude that the ASEBA derived proxy scales are valid and can be used reliably in the current and future research.

### Heritability of the proxy measure of Type D personality

Table 1 describes the composition of the NTR sample that provided dichotomous Type D and continuous NA and SI subcomponent data. The Type D dataset consisted of 30,433 individuals in 11,106 extended families. MZ and DZ twins accounted for more than half of the individuals in the pedigree (N_MZ_ = 7380/N_DZ_ = 8810), with more female than male twins (MZ males vs. females: 2389 vs. 4991; DZ males vs. females: 3479 vs. 5331). In total, 4159 spouse pairs were included: 2416 pairs (N_twin-spouse pair_^1^ = 155) with offspring and 1743 pairs (N_twin-spouse pair_ = 1394) without offspring. The datasets for the NA and SI subcomponents showed very similar structures (Table 1). The prevalence of Type D personality in the NTR sample was 21%.

**Table 1:** Number and type of NTR participants that provided Type D and NA/SI subcomponent data

	Type D pedigree	NA pedigree	SI pedigree
Number of extended families	11,106	11,205	10,843
Individuals	30,433	30,857	29,685
MZ male (individuals)	2389	2411	2330
MZ female (individuals)	4991	5051	4916
MZ (individuals)	**7380**	**7462**	**7246**
DZ male (individuals)	3479	3507	3348
DZ female (individuals)	5331	5440	5218
DZ (individuals)	**8810**	**8947**	**8566**
Unknown zygosity male (individuals)	90	92	89
Unknown zygosity female (individuals)	169	168	164
Unknown zygosity (individuals)	**259**	**260**	**253**
Fathers	3019	3087	2894
Mothers	4076	4183	3925
Parents	7095	7270	6819
Brothers	1635	1644	1601
Sisters	2795	2826	2772
Full sibs (non-twin individuals)	**4430**	**4470**	**4373**
Half-brothers	28	29	28
Half-sisters	44	44	46
Half-sibs (non-twin individuals)	**72**	**73**	**74**
Spouse pairs with offspring	2416 pairs	2486 pairs	2320 pairs
Spouse pairs without offspring	1743 pairs	1747 pairs	1739 pairs
Grandmother/grandfather/granddaughter/grandson	75 pairs	74 pairs	75 pairs
Avuncular (Aunt/uncle/niece/nephew)	1984 pairs	1989 pairs	1961 pairs
First cousin	544 pairs	549 pairs	544 pairs
First cousin, MZ twins as parents	70 pairs	70 pairs	70 pairs
Double first cousin	1 pairs	1 pairs	1 pairs

The sums of male-specific and female-specific count (i.e. total counts by adding males and females) are given in bold

Data are presented as N, unless otherwise indicated

To summarize the familial resemblance for NA, SI, NAxSI, and Type D personality, pairwise correlations were estimated for the various relationships (see Table 2). In general, correlations were highest for MZ twins in all measures, with MZ correlations being always more than twice as high as the DZ twin correlations. Correlations were slightly stronger for DZ twin than for sibling pairs in NA, NAxSI and dichotomous Type D measures, and a similar patterns was observed for SI (DZ vs. siblings: 0.14 vs. 0.15). Parent–offspring correlations showed a consistent pattern that mother-daughter correlations were larger than correlations from the other three parent–offspring relationships. The spouse correlations in parents of twins were larger than the correlations between twins and their own spouses, who were on average 15 years younger than the twins’ parents, indicating a longer household sharing may increase the resemblance on Type D and its subcomponents through marital interaction. Together with the low magnitude of the spouse correlation, this suggests little phenotypic assortment for Type D personality.

**Table 2:** Basic summary of familial correlations and their confidence intervals for NA, SI, the continuous NAxSI Type D measure, and the historic dichotomous Type D classification

	NA			SI			NAxSI			Type D dichotomous		
	N	Mean age	Correlation (95%CI)	N	Mean age	Correlation (95%CI)	N	Mean age	Correlation (95%CI)	N	Mean age	Correlation (95%CI)
MZ twins	3187	30.1	0.46 (0.43, 0.48)	3093	30.3	0.44 (0.41, 0.47)	3083	30.3	0.41 (0.38, 0.44)	3144	30.2	0.48 (0.43, 0.53)
MZ male	1011	28.1	0.41 (0.36, 0.46)	977	28.2	0.45 (0.40, 0.50)	974	28.2	0.38 (0.33, 0.43)	999	28.2	0.45 (0.33, 0.55)
MZ female	2176	31.1	0.44 (0.41, 0.47)	2116	31.3	0.44 (0.40, 0.47)	2109	31.3	0.41 (0.37, 0.44)	2145	31.2	0.46 (0.40, 0.53)
DZ twins	3382	26.3	0.18 (0.15, 0.21)	3233	26.6	0.14 (0.11, 0.18)	3216	26.6	0.13 (0.10, 0.16)	3322	26.4	0.22 (0.17, 0.28)
DZ males	661	24.8	0.15 (0.08, 0.23)	640	25.1	0.10 (0.02, 0.18)	633	25.1	0.09 (0.01, 0.17)	657	24.9	0.12 (-0.04, 0.29)
DZ females	1186	28.0	0.16 (0.10, 0.22)	1133	28.4	0.20 (0.14, 0.25)	1132	28.4	0.16 (0.10, 0.22)	1158	28.2	0.23 (0.15, 0.32)
DZ opposite sex	1535	25.5	0.20 (0.15, 0.25)	1460	25.8	0.12 (0.06, 0.17)	1451	25.8	0.11 (0.06, 0.16)	1507	25.6	0.24 (0.14, 0.33)
Sibling-Sibling	1729	37.2	0.11 (0.06, 0.16)	1715	37.5	0.15 (0.11, 0.20)	1711	37.4	0.10 (0.05, 0.14)	1722	37.3	0.15 (0.05, 0.24)
Father-son	1966	57.2/ 22.7	0.13 (0.08, 0.17)	1827	57.5/ 23.0	0.09 (0.05, 0.14)	1813	57.5/ 23.0	0.06 (0.02, 0.11)	1919	57.3/ 22.8	0.07 (-0.04, 0.17)
Father-daughter	3014	56.9/ 23.4	0.11 (0.07, 0.14)	2807	57.2/ 23.7	0.12 (0.08, 0.16)	2790	57.1/ 23.7	0.09 (0.05, 0.13)	2933	57.0/ 23.5	0.15 (0.07, 0.23)
Mother-son	2501	54.9/ 22.6	0.14 (0.11, 0.18)	2306	55.2/ 22.9	0.15 (0.11, 0.19)	2295	55.2/ 22.9	0.10 (0.06, 0.14)	2435	55.0/ 22.7	0.10 (0.02, 0.18)
Mother-daughter	4.053	54.4/ 23.4	0.17 (0.13, 0.19)	3792	54.8/ 23.7	0.17 (0.14, 0.20)	3771	54.8/ 23.7	0.15 (0.12, 0.18)	3916	54.6/ 23.5	0.21 (0.15, 0.26)
Parents of twins	4117	54.4/ 56.6 (Mo/Fa)	0.12 (0.09, 0.15)	3815	54.8/ 56.9 (Mo/Fa)	0.10 (0.07, 0.13)	3787	54.7/ 56.9 (Mo/Fa)	0.10 (0.07, 0.14)	3983	54.6/ 56.7 (Mo/Fa)	0.17 (0.11, 0.22)
Twin-spouse	1577	37.5/ 40.2	0.07 (0.02, 0.12)	1577	37.3/40.2	0.13 (0.08, 0.18)	1575	37.3/ 40.2	0.08 (0.03, 0.13)	1579	37.4/ 40.2	0.13 (0.04, 0.22)

*N* the number of complete pairs, (*Mo*/*Fa*) (Mother/Father)

The additive genetic, non-additive (dominance) genetic, and shared household variance component estimates and their standard errors are given in Table 3, with the log-likelihood (LL) for each model and p-values derived from the likelihood ratio test. In all three Type D associated measures, removing either age or sex led to a worse fitting model. Dropping the A, D or H component from the full ADHE model led to a worse fit.

**Table 3:** Parameter estimates (and SE) for variance components

Model	∆df	Log-likelihood	χ^2^ (p-value)	Estimates of variance components (standard error)					Broad-sense heritability (standard error)^b^
				A	D	H	E	Total variance	
NA									
ADE plus^a^ household	–	− 11,422.35	–	0.11 (0.014)	0.28 (0.013)	0.066 (0.010)	0.34 (0.014)	0.79 (0.026)	0.49 (0.011)
ADE no household	1	− 11,444.53	44.36 (< .001)	0.12 (0.013)	0.28 (0.013)	–	0.39 (0.012)	0.79 (0.022)	0.51 (0.011)
AE plus household	1	− 11,633.95	423.2 (< .001)	0.24 (0.014)	–	0.064 (0.010)	0.49 (0.016)	0.79 (0.023)	0.30 (0.014)
SI									
ADE plus^a^ household	–	− 11,870.74	–	0.099 (0.015)	0.32 (0.014)	0.066 (0.011)	0.36 (0.015)	0.84 (0.028)	0.50 (0.011)
ADE no household	1	− 11,891.79	42.1 (< .001)	0.12 (0.014)	0.32 (0.014)	–	0.40 (0.013)	0.84 (0.024)	0.52 (0.011)
AE plus household	1	− 12,099.76	458.04 (< .001)	0.23 (0.016)	–	0.068 (0.011)	0.54 (0.017)	0.84 (0.026)	0.27 (0.014)
NAXSI									
ADE plus^a^ household	–	− 10,297.09	–	0.093 (0.014)	0.28 (0.012)	0.066 (0.010)	0.32 (0.014)	0.76 (0.025)	0.49 (0.012)
ADE no household	1	− 10,320.26	46.34 (< .001)	0.11 (0.013)	0.28 (0.012)	–	0.37 (0.012)	0.76 (0.021)	0.51 (0.011)
AE plus household	1	− 10,523.61	453.04 (< .001)	0.22 (0.015)	–	0.068 (0.010)	0.48 (0.016)	0.76 (0.024)	0.28 (0.015)

∆*df* the difference of degree of freedom; *χ*^2^ (p-value): likelihood ratio test statistic calculated by − 2 (log likelihood sub-nested model – log likelihood full model); *p-value* log-likelihood ratio test p-values; *A* additive genetic variance; *D* dominance genetic variance; *H* shared household variance; *E* non-genetic variance

^a^The final selected model based on ∆χ^2^

^b^Broad-sense heritability column gives the proportion of variance explained by additive and dominance genetic variance

From the full models, the broad-sense heritability for continuous NA, SI and NAxSI was estimated as 49%, 50% and 49% respectively, with substantial contributions from genetic dominance (NA: 0.28/0.79 = 35%; SI: 0.32/0.84 = 38%; NAxSI: 0.28/0.76 = 37%) (Table 3). Household effects showed small effects with average contributions of ~ 8% to the total phenotypic variance.

To estimate the genetic and environmental correlations between NA and SI, a bivariate variance decomposition analysis was performed and the model fitting results are shown in Table 4. Compared with the full model, removing either shared household effects (H) and/or non-additive genetic effects (D) led to a worse fitting model. The phenotypic correlation between NA and SI in the current study population was 0.51 and phenotypic covariance between NA and SI was 0.42 (SE = 0.020). A/D/H/E components contributed respectively 13.6%, 49.6%, 15.2%, and 21.6% to the total phenotypic correlation. The genetic correlations were *r*_A_ = 0.57 for additive and r_D_ = 0.69 for non-additive factors. Taking both additive and non-additive genetic factors, the genetic correlation was 0.66. Although shared household effects revealed a high correlation between the two subcomponents of Type D (*r*_H_ = 0.94), they only explained 15.2% of the phenotypic correlation due to limited main shared household effect on NA and SI.

**Table 4:** Bivariate genetic model fitting results for negative affectivity and social inhibition

Number	Model	∆df	log-likelihood	Vs	χ^2^	χ^2^ p-value
1	Full NA (ADEH_NA_) and SI (ADEH_SI_)*	–	− 18,359.92		–	–
2	ADE_NA_–ADE_SI_	3	− 18,396.53	1	73.2	< .001
3	AE_NA_–AE_SI_	6	− 18,758.57	1	797.4	< .001

*ADEH*_*NA*_ models including additive, dominant, share environmental and household effects for negative affectivity; *ADEH*_*SI*_ models including additive, dominant, share environmental and household effects for social inhibition; ∆*df* the difference of degree of freedom; *Vs*. compare with the model; *χ*^2^ likelihood ratio test statistic calculated by − 2 (log likelihood sub-nested model – log likelihood full model); χ^2^ p-values

*Selected model with the best fitting

## Discussion

In the current study, an ASEBA-based proxy measure of Type D personality was constructed with six items assessing NA and six items assessing SI. The proxy scale was extensively validated, and demonstrated good construct validity, reliability, and high convergent validity against the gold standard measure of Type D (DS14), and related constructs such as neuroticism, depression, introversion, and anxiety. In addition, the proxy scale was measurement invariant with respect to age and sex, except for the latent factor means. Employing the proxy scales, an extended twin-pedigree analysis of 30,433 individuals rendered high broad-sense heritability estimates for NA (49%), SI (50%), and the continuous Type D personality measure (49%). Household effects showed small but significant contributions (4–9%) to the total phenotypic variation. The significant additive (0.57) and non-additive (0.69) genetic correlation between NA and SI indicated a shared gene set influencing both pillars of Type D personality. Household effects were highly correlated and of similar size for NA and SI.

Identifying the genetic variants underlying the heritability of Type D personality as found in the current analysis could help understand the pathophysiological pathways leading to its well-known association with cardio-metabolic endpoints through Mendelian Randomization (Davey Smith and Hemani 2014). This requires large-scale studies and/or collaborations across many cohorts that measured Type D personality, in addition to genome-wide typing of genetic (single nucleotide polymorphisms) markers so as to allow genome-wide association studies. Currently, only a limited number of such genetically informative cohorts have included the DS14 to measure Type D. Proxy measures could help increase the number of cohorts that include Type D assessment greatly. In 2007, Kupper and colleagues developed a Type D proxy in the NTR by applying the “combination of scales” method, with 20 items being selected from three questionnaires (including some ASEBA items) in different measurement scales (Kupper et al. 2007). However, these items are not available together in many other cohorts, or even in all NTR surveys. Therefore, we adopted another often used instrument, i.e. ASEBA, to construct a new proxy for Type D personality, with all items belonging to a single questionnaire, being quantified in the same measurement scale, and with repeated measurements in the NTR to maximize the sample size.

Previous studies using structural equation modeling in a classical twin design on Type D personality estimated the heritability of dichotomous Type D classification to be between 34 and 52%, depending on the time-point of measurements (Kupper et al. 2011, 2007). Here, our heritability estimate for a continuous measure for Type D (i.e. the NAxSI interaction) was at the high end of this interval, 49% (95%CI 47–51%). Additionally, the tetrachoric correlation of Type D dichotomy was 0.48 (95%CI 0.43–0.53) for MZ twins, which is also in line with the previous findings on the heritability of dichotomous Type D classification.

We found broad-sense heritability estimates for NA of 49% and for SI of 50% with a substantial non-additive contribution to both subcomponents of Type D. Because the classical twin design does not allow simultaneous estimations of the contribution of genetic non-additive (D) factors and common environmental (C) factors, it constrains C to be zero when an ADE model is evaluated. In theory, the violation of the ‘C = 0′ assumption should lead to an overestimation of additive genetic components while underestimation of non-additive genetic components (Keller et al. 2010). This is perfectly in line with the contrast between the observations of the current extended twin-family study and that of our previous classical twin study, where broad-sense heritability for SI and NA were 40–45% and 42–49% (Kupper et al. 2011), but where significant non-additivity was detected for SI but not for NA. It should be noted though that the major contribution of non-additive genetic effects may have implications for genome-wide association studies (GWAS), as a GWAS typically assumes additive, rather than non-additive, genetic effects.

Household effects on Type D were estimated to be small, but significant in the current study. For related phenotypes such as neuroticism (Boomsma et al. 2018) household effects have been estimated, with a similar size. The household effect correlated perfectly between NA and SI, and prolonged household sharing appears to increase the resemblance on NA, SI and Type D personality in spouses. This suggests that the Type D personality characteristics (NA, SI or both) of one person in a household may constitute an environmental influence of importance for another household member, affecting their Type D characteristics, e.g., through marital interaction or by parental modeling. The overall substantial role of unique environmental factors in explaining variance in Type D personality characteristics holds promise for the development of psychological interventions, which may address improving regulatory skills at the cognitive, behavioral and emotional level.

As a weakness we note that the likelihood-ratio test of a variance component does not follow the usual central χ^2^(1) distribution under the hypothesis that the variance component is zero, which may have inflated the p-value estimations. However, the difference between the log-likelihoods from full model to reduced models were substantially larger than zero. Another limitation is that we were not able to model the genetic effects of phenotypic assortative mating, as this is not possible in Mendel. Strengths of the study comprised the employment of an extended pedigree design, and the use of an extensively validated proxy measure of Type D personality.

## Conclusions

The ASEBA-based proxy measure of Type D personality is valid and reliable. Extended pedigree analysis of the ASEBA-based proxy confirmed that Type D personality and both NA and SI subcomponents are substantially heritable, with a shared genetic basis for the subcomponents. These results warrant further exploration of the genetic variants underlying this heritability, which in turn can be used to investigate the biological pathways underlying the risks of cardiovascular disease incidence and progression associated with Type D personality.

## Electronic supplementary material

Below is the link to the electronic supplementary material.

Supplementary file1 (DOCX 69 kb)

Supplementary file2 (DOCX 271 kb)

## Footnotes

1 A twin-spouse pair correlation refers to a twin correlation with his or her own spouse. A spouse pair correlation being a correlation between the parents of the twins.
