# Supplementary material for: The Heritability of Type D Personality by an Extended Twin-Pedigree Analysis in the Netherlands Twin Register
Source: Behav Genet. 2020 Oct 16;51(1):1–11. doi: 10.1007/s10519-020-10023-x (PMC7815549; doi:10.1007/s10519-020-10023-x)
Supplement: Supplementary file 1 — Supplementary file1 (DOCX 69 kb) [file 10519_2020_10023_MOESM1_ESM.docx]

Supplement – Methods and results of proxy validation analysis

# Methods

Three groups of participants took part in a validation study of the ASEBA-based proxy for Type D personality. The data from one group of participants (student sample) were analyzed to explore the factor structure, while in the second (general population sample) we confirmed the factor structure, and examined measurement invariance and construct validity. The third sample was derived from the NTR pedigree dataset, and was used to test whether the same results apply in this NTR pedigree subgroup of unrelated individuals (i.e. parents of twins) as compared to the general population.

## Samples

Sample 1: Undergraduate students – We approached an undergraduate student sample from Tilburg University (N=214, 91% women, 87% European descent) for initial validation. Students filled out a psychological survey around December 2018 in exchange for course credits.

#### Materials

*Questionnaires –* A selection of items from the adult ASEBA scale that resembled the items from the DS14 in content and wording (Achenbach & Rescorla, 2003), and the DS14 as a gold standard for measuring Type D personality (Denollet, 2005) were administered.

Sample 2: General population – A general population sample was recruited through convenience sampling and snowball recruiting, as part of a student research project (four groups of eight students approached acquaintances, friends of their families, and unfamiliar people, and asked them to participate in an anonymous survey on *Personality & Your health*). Sampling was structured such that for each decade between 20 and 90, an equal number of people were asked to participate, with an equal sex distribution in each decade. Of all participants who provided an email address (N=688), eight (1%) refused participation. There were no exclusion criteria. This resulted in a sample of in total 680 people (55% women, average age = 49 ± 16), whose data were analyzed to further validate the ASEBA-based proxy.

#### Materials

For the current analyses, we used the following questionnaires of the Sample 2dataset. With respect to personality, we assessed neuroticism and extraversion with the Big Five Inventory (Neuroticism (8-items), Extraversion (8 items); 5-point Likert scale; Denissen et al., 2008). With respect to emotional functioning, we assessed depressive symptoms (PHQ-9 (4-point Likert scale; Kroenke, Spitzer, & Williams,2001)) and anxiety symptoms (GAD-7 (4-point Likert scale; Spitzer, Kroenke, Williams, & Löwe, 2006)). The twelve items from the Adult Self Report ASEBA scale (6 negative affectivity (NA), 6 social inhibition (SI); 3-point Likert scale; Achenbach & Rescorla, 2003) selected based on the analysis in sample 1 were assessed, as well as the DS14 (14 items, 7 NA and 7 SI; 5-point Likert scale) as a gold standard assessment for Type D personality (Denollet, 2005).

Sample 3: Parent subset of NTR pedigree dataset – We selected the parents from the NTR pedigree dataset (see main text) to confirm factor structures found in the other two datasets, and to assess measurement invariance with respect to age and sex. This subset consisted of 7936 parent dyads (50% women, average age of 49.1 ± 12.1).

#### Materials

We analyzed the 12 ASEBA-based proxy items (6 NA, 6 SI; 3-point Likert scale; Achenbach & Rescorla, 2003) as well as age and sex for this analysis.

## Statistical analysis

Sample 1: To examine whether the ASEBA-based proxy captured the same facets as the DS14, principal components analysis and reliability analysis were performed to assess the internal structural validity of the proxy scale. We started by examining the factorability of the data, by gauging the Kaiser-Mayer-Olkin (KMO) statistic. In the principal components analysis, we choose principal axis factoring, with Oblimin rotation to account for the correlation between the proxies of NA and SI.. For the extraction of the number of factors, the eigenvalue > 1 rule was applied. Then, a higher order factor analysis was performed on the same samples with the continuous ASEBA derived NA and SI sumscores, together with the original NA and SI sum scores based on the DS14.

Individuals with both high NA and SI (≥10 on both the DS14 subscales) were classified as Type D personality. The dichotomous cut-off for Type D personality by the ASEBA proxy scale was determined by maximizing Type D personality classification consistency between DS14 and ASEBA proxy scale in the contingency table.

Sample 2: Construct validity of the ASEBA proxy was assessed in several ways. McDonalds’ Omega (McDonalds, 1999) was calculated in R to assess internal consistency (R version 3.4.2; R development core team, 2008; psych package). Then, a confirmatory factor analysis (CFA; lavaan package; (version 0.5-23.1097; Rosseel, 2012)) was performed including the 14 original DS14 items and the 12 proxy ASEBA items, with four latent factors (two for the DS14 (NA, SI) and two for the proxy (Proxy NA, Proxy SI)). The CFA used Weighted Least Squares estimation with Missing Values (WLSMV). We fit a model for ordinal data with missing values set at pairwise deletion (Rhemtulla, Brosseau-Liard, & Savalei, 2012). The variance of the latent factors was fixed at 1. We tested a correlated four-factor model, for which we hypothesized that the DS14 NA and SI factors would correlate highly with the ASEBA proxy NA and SI factors. Then, we tested whether a correlated 2-factor model would fit the data better, assuming similarity between DS14 derived NA and SI and ASEBA derived NA and SI, respectively.

For the ASEBA-based scales we then assessed measurement invariance for four age categories (based on 25^th^(35), 50^th^ (50), and 75^th^ (61) percentile) and sex (two categories) using the SemTools package (version 0.5-2; Jorgensen, Pornprasertmanit, Schoemann, & Rosseel, 2019), to establish if any covariate effects in the heritability analyses were not due to scale artifacts. The measEq.syntax command in SemTools examines accumulative measurement invariance at three levels compared to the configural (equal factor structure) model: equal factor loadings (metric invariance), equal item thresholds (scalar invariance), and equal latent factor means (means invariance). We tested thresholds instead of intercepts because of the categorical nature of the items loading on the latent factors. The data were thus defined as ordered, indicating an ordinal measurement scale of the items. See footnote for an example of the R syntax for the configural model and the equal factor loadings model ^[[1]](#footnote-1)^. When comparing latent means between groups, the residuals model can be skipped, which is why we continued with the equal latent means model (Putnick & Bornstein, 2016). For the nested models, we display the LRT, CFI and RMSEA and their deltas for fit comparison. Because in large samples, the LRT is very sensitive to small changes in measurement variance, we decided to follow Meade, Johnson, & Braddy, (2008) and base model comparison on the change in CFI and RMSEA. Research has shown that these are valid model fit comparison measures, with a .01 reduction in CFI and/or RMSEA fit indicating a significant worsening of fit, which would suggest measurement variance at that level (Meade, Johnson, & Braddy, 2008).

In SPSS, we examined construct validity further by calculating Pearson correlations of the continuous gold standard (DS14) and proxy NA and SI scales with continuous neuroticism, extraversion, anxiety, and depression scales. Spearman correlations were calculated between these latter scales and the dichotomous Type D classifications (gold standard and proxy).

To assess sensitivity, specificity, positive predictive value, and negative predictive value, we calculated crosstabs for the ASEBA derived (cut-off = 3 on NA proxy and SI proxy, initially derived in sample 1) and gold standard Type D (cut-off = 10 for both scales) classifications. Finally, we calculated a receiver operator curve (ROC) for the ASEBA derived continuous Type D variable (proxy NA x proxy SI) predicting the gold standard classification of Type D (DS14, 1 = positive status). The area under the curve was calculated as a measure of diagnostic accuracy.

Sample 3: McDonalds Omega was calculated to assess internal consistency of the ASEBA based proxy scales. A confirmatory factor analysis was then performed in R in the lavaan package (version 0.5-23.1097; Rosseel, 2012). We tested a correlated 2-factor model, and evaluated its fit. Then we did a measurement invariance analysis (measEq.syntax) for age categories (until 30, until 60 and until 90) and sex (version 0.5-2; Jorgensen, Pornprasertmanit, Schoemann, & Rosseel, 2019). For details, see methods section of sample 2.

# Results

## Sample 1

Exploratory factor analysis on DS14 and ASEBA derived items – The Kaiser-Mayer-Olkin (KMO) measure for sampling adequacy was 0.87, suggesting good factorability of the data. Principal axis factoring with Oblimin rotation to account for the correlation between NA and SI resulted in a clear two-factor structure for which the rotated factor loadings are presented in Table S1 (only shows factor loadings larger than .20). Visualized loadings ranged between .34 and .70 for the SI proxy and between .57 and .75 for the NA proxy. There were two cross loadings, one for each construct. NA and SI proxies correlated .55 with each other.

The correlations of SI and NA subscales between DS14 and ASEBA proxy scales were both .80. Higher order factor analysis of the sum scores of the original and proxy scales rendered a two-factor solution with both NA scales loading on one factor (loadings: .85 each) and both SI scales loading on the other factor (loadings: .85 each). With respect to internal consistency, the six items of the SI proxy obtained a Cronbach’s alpha of .74, while a Cronbach’s alpha of .83 was observed for the NA proxy. Item-total correlations were between .28 and .67 for the 12 proxy items, with median correlations of .50 and .61 respectively for the SI and NA proxies, indicating a high level of internal consistency of the selected proxy questions within these two factors. Descriptive statistics are presented in Table S2.

Cut-off validity - Participants in the validation study scoring high on both NA and SI proxies, with a cut-off ≥3 were classified as Type D personality. Prevalence in this student sample was 44% using the gold standard and 48% using the proxy classification. This prevalence is typical amongst first year undergraduate students who experience a lot of social stress due to their transition to student life, often living on their own for the first time in their lives. The Type D personality identification achieved an optimal consistency between original DS14 classification and the ASEBA proxy scale classification using the cut-off of ≥3. At this cut-off, sensitivity was 87%, and so was specificity. Positive predictive value was 84% and negative predictive value 90%, which is considered very good.

Internal consistency - We used R to calculate McDonald’s Omega to gauge internal consistency of the ASEBA derived NA and SI scales in the student sample. Omegas are listed in Table S3, and show excellent internal consistency.

**Table S1. Factor structural validity and internal consistency of ASEBA derived NA and SI scales (N=214)**

|  | ASEBA item | Item-total correlation (*r)* | Cronbach’s alpha if item deleted | Component 1 | Component 2 |
| --- | --- | --- | --- | --- | --- |
| Social inhibition items | | | | | |
| 1 | ASEBA75 – Too shy or timid | .45 | .71 |  | .54 |
| 2 | ASEBA67 – Trouble making & keeping friends | .58 | .66 |  | .70 |
| 3 | ASEBA69 – Reserved, keep things to self | .52 | .69 |  | .61 |
| 4 | ASEBA42 – Rather be alone than with others | .55 | .67 |  | .66 |
| 5 | ASEBA111 – keep from getting involved with others | .47 | .70 |  | .56 |
| 6 | ASEBA 48 – Not liked by others | .28 | .74 | *.34* | .34 |
| α=0.74 | | | | | |
| Negative affectivity items | | | | | |
| 1 | ASEBA103 – I am unhappy, sad, or depressed | .59 | .81 | .66 | *.51* |
| 2 | ASEBA112 – I worry a lot | .63 | .80 | .71 |  |
| 3 | ASEBA50 – I am too fearful or anxious | .61 | .80 | .70 |  |
| 4 | ASEBA45 – I am nervous or tense | .67 | .79 | .75 |  |
| 5 | ASEBA115 – I feel restless | .60 | .80 | .67 |  |
| 6 | ASEBA87 – Moods and feelings change suddenly | .52 | .82 | .57 |  |
| α=0.83 | | | | | |

**Note:** factor loadings are listed in the final two columns when they were larger than .20.

## Sample 2

On average, sample 2 was 49 (SD=16) years of age, and 55% were female. The sample was relatively highly educated, with only 13% having completed high school or less. Type D prevalence was 23% in this general population sample according to the gold standard and 21% according to the ASEBA proxy. Descriptive statistics are presented in Table S2.

**Table S2. Descriptive statistics for the three samples**

|  | Student sample | General population sample | Twin pedigree parental sample |
| --- | --- | --- | --- |
| Age (*Mean, SD*) | 20 (2) | 49 (16) | 49 (12) |
| Sex *(% women, N)* | 91% (195) | 55% (372) | 50% (3968) |
| Education *(% high school or less, N)* | 100% (214) | 13% (90) | - |
|  |  |  |  |
| DS14 – NA scale (*Mean, SD*) | 11.5 (5.3) | 8.1 (4.7) | - |
| DS14 – SI scale (*Mean, SD*) | 10.9 (5.8) | 9.4 (5.2) | - |
| Type D DS14 (*%, N*) | 44% (94) | 23% (154) | - |
| ASEBA proxy NA scale | 4.5 (3.0) | 2.3 (2.1) | 1.9 (2.1) |
| ASEBA proxy SI scale | 3.2 (2.3) | 2.3 (2.5) | 2.0 (1.9) |
| Type D ASEBA (*%, N*) | 46% (104) | 21% (147) | 17% (1335) |
|  |  |  |  |
| Depressive symptoms score (*Mean, SD*) | - | 2.9 (3.6) | - |
| Anxiety symptoms score (*Mean, SD*) | - | 2.7 (3.5) | - |
| Neuroticism score (*Mean, SD*) | - | 2.6 (0.7) | - |
| Extraversion score (*Mean, SD*) | - | 3.5 (0.6) | - |

Internal consistency – McDonalds’ omegas calculated for the general population sample showed excellent internal consistency (Table S3).

**Table S3. McDonalds Omegas for the three samples**

| Sample | Omega total - total scores | Omega total – NA proxy | Omega total – SI proxy |
| --- | --- | --- | --- |
| Students | .91 | .89 | .84 |
| General population | .93 | .92 | .83 |
| Parent dyads NTR | .92 | .83 | .90 |

CFA – In a confirmatory factor analysis of the general population sample data, we first tested a four-factor model (including DS14 and proxy scales), and correlated two two-factor models. We left out the cross-loadings, because we wanted to compare the fit with the original DS14 structure, and stay closest to this structure for the proxy.

In the correlated 4-factor model, the correlation between the latent proxy NA and the latent DS14 NA factors was .91. For SI, the between-factor correlation was .86. Cross-correlations laid between .41 and .61 respectively. The next step was to add the ASEBA derived and DS14 derived SI items together in one factor and do the same for the NA items. Testing this 2-factor model showed that this model fitted the data well (because of the sample size, we are ignoring the chi-squared difference test). Then, we moved all the DS14 items out, and examined the correlated 2-factor model for the ASEBA items only. Fit indices TLI and RMSEA indicated that the fit of the correlated 2-factor model was excellent (Table S4).

**Table S4. Model fit for CFA of ASEBA based proxy items in the general population sample**

| Model | Free Par | Df | Chi square | Δ Chi square | Δ df | Significance | Robust fit indices | | |
| --- | --- | --- | --- | --- | --- | --- | --- | --- | --- |
|  |  |  |  |  |  |  | **χ^2^ / df** | **TLI** | **RMSEA** |
| ASEBA and DS14 |  |  |  |  |  |  |  |  |  |
| Correlated 4-factor model |  | 293 | 1299.76 | - | - | - |  | .95 | .072 |
| Correlated 2-factor model (all items) |  | 298 | 1438.142 | 138.382 | 5 | <.000 | 4.83 | .95 | .076 |
| ASEBA only |  |  |  |  |  |  |  |  |  |
| Correlated 2-factor | 37 | 53 | 268.35 | - | - |  | 5.06 | .94 | .078 |

Measurement invariance – We examined measurement invariance for age and sex. The results from the measurement invariance analysis for age categories (Table S5) showed that that the fit hardly changed due equalizing loadings and thresholds across age groups. Equalizing the means induced a significant drop in fit (ΔCFI>.01), indicating that item means were different between age groups. With respect to sex, the modeling showed a similar pattern, with equal factor loadings and thresholds for men and women, but different item means.

**Table S5. Measurement invariance results for the general population sample**

|  | Model | df | Chi square | Fit indices | | | Deltas | |
| --- | --- | --- | --- | --- | --- | --- | --- | --- |
|  |  |  |  | **CSDT** | **CFI** | **RMSEA** | **ΔCFI** | **ΔRMSEA** |
| Age | Equal factor structure (configural invariance) | 212 | 418.268 | - | .948 | .077 | - | - |
|  | Factor loadings (metric invariance) | 242 | 438.178 | .919 | .950 | .070 | .002 | .007 |
|  | Thresholds (scalar invariance) | 264 | 445.732 | .998 | .948 | .071 | .000 | .000 |
|  | Means (means invariance) | 275 | 524.095 | **<.001** | .937 | .074 | **-.011** | .004 |
| Sex | Equal factor structure (configural invariance) | 106 | 270.203 | - | .962 | .068 |  |  |
|  | Factor loadings (metric invariance) | 116 | 289.645 | *.035* | .960 | .067 | .002 | .001 |
|  | Thresholds (scalar invariance) | 132 | 274.457 | 1.00 | .963 | .064 | .003 | .003 |
|  | Means (means invariance) | 144 | 421.779 | **<.001** | .932 | .083 | **-.31** | **-.021** |

Note: CSDT – Chi square difference test. Please note that while the metric invariance model for sex showed a significant worsening of fit in the chi square difference test, it did not in the delta CFI and delta RMSEA tests. Because of the large sample size for which the CSDT is sensitive, we followed the latter fit indices difference tests (Meade et al. 2012).

Construct validity – In SPSS, we calculated an intra-class correlation (ICC) to assess test-retest reliability of the two NA (original and proxy) and two SI (original and proxy) scales, respectively. Results showed that the ICC between the NA scales was .76 and .65 between the SI scales. ICC’s between .60-.80 indicate good agreement between measures.

We then computed Pearson correlations to relate the continuous DS14 and ASEBA derived scales with the continuous neuroticism, extraversion, depression, and anxiety scales. Results showed that the ASEBA NA and SI scales correlated with personality and mood in a similar way, compared to the DS14 NA and SI scales (Table S6), although there were some notable differences in the size of the correlation, sometimes differing >.10. This might have to do with the difference in the scale range (3-point vs. 5-point Likert scale), which restricts the variance of the ASEBA scales compared to the DS14 scales.

**Table S6. Construct validity comparison**

|  | Neuroticism | Extraversion | Depression | Anxiety |
| --- | --- | --- | --- | --- |
| DS14 NA scale | .78** | -.40** | .55** | .60** |
| ASEBA NA scale | .73** | -.31** | .65** | .72** |
| DS14 SI scale | .37** | -.75** | .28** | .23** |
| ASEBA SI scale | .34** | -.66** | .36** | .32** |
| DS14 Type D | .48** | -.46** | .37** | .40** |
| ASEBA Type D | .48** | -.43** | .39** | .41** |

Sensitivity and specificity of threshold – Cross-tabs (Table S7) were calculated for sensitivity analysis (i.e., the probability that the proxy will indicate Type D personality among those with Type D personality according to the gold standard) and specificity (i.e., the proportion of those without Type D personality according to the proxy who will indeed have a negative test result based on the gold standard) of the proxy scale cut-off in comparison to the gold standard (DS14 cut-off). Comparing the most optimal trade-off between sensitivity and specificity, a cut-off at 3 was deemed optimal (as was determined to be optimal in sample 1). The placement of the cut-off at 3 resulted in a similar prevalence of Type D in the proxy and the gold standard (See Table S2).

Specificity of the proxy scale was 91%. Sensitivity was lower, at 63%. From a clinician’s point of view, positive predictive value (PPV, what is the chance that a person with a positive proxy result truly has Type D?) and negative predictive value (NPV, what is the chance that a person with a negative proxy result truly does not have Type D?) are of interest. PPV of the proxy scale was 68%, while NPV was 89%.

**Table S7 Crosstabs of Type D classifications**

|  | | Type D | |
| --- | --- | --- | --- |
|  |  | NO | YES |
| Type D proxy | NO | 475 | 56 |
|  | YES | 45 | 96 |

^1^ true negatives, ^2^ false positives, ^3^ false negatives, ^4^ true positives

ROC curve – We calculated a receiver-operator curve (ROC) for the interaction of the ASEBA derived NA and SI scales (continuous Type D variables) predicting the gold standard classification (dichotomous DS14 derived Type D classification). The ROC is plotted in Figure S1 below. Results show that the area under curve, which summarizes the diagnostic accuracy of the proxy, was high, at .86 (95% CI = .82-.90), suggestive of a good diagnostic instrument.

**Figure S1. ROC curve for continuous NA x SI variable predicting gold standard Type D classification**


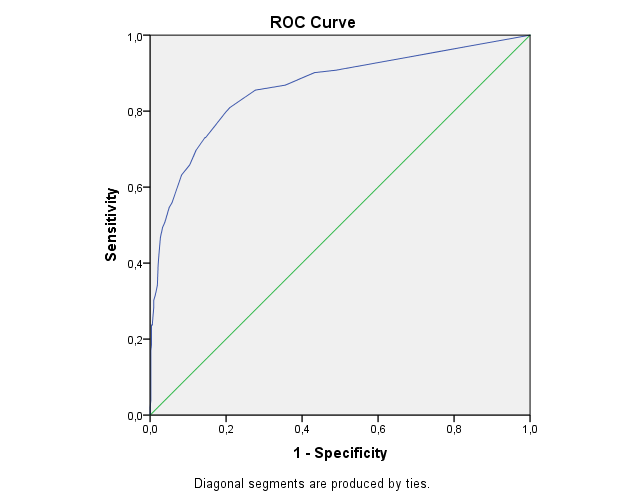


## Sample 3

Internal consistency - In the NTR data subset (Descriptive statistics are presented in Table S2), Omega total was .92 for the general factor, .90 for the social inhibition proxy subscale and .83 for the negative affectivity proxy subscale, suggesting excellent internal consistency of the scale (Table S3).

CFA – We repeated the confirmatory factor analysis in the NTR data subset. Results showed that a correlated two-factor model provided an excellent fit to the data, compared to the orthogonal model, in which factors were constrained to be uncorrelated. Fit indices TLI and RMSEA indicated that the fit of the correlated 2-factor model was excellent (Table S8).

**Table S8. Model fit for CFA of ASEBA based proxy items in NTR data subset**

| Model | df | Chi square | Δ Chi square | Δ df | Significance | Robust fit indices | | |
| --- | --- | --- | --- | --- | --- | --- | --- | --- |
|  |  |  |  |  |  | **χ^2^ / df** | **TLI** | **RMSEA** |
| Orthogonal 2-factor | 54 | 11814.313 | - | - |  | 218.78 | .62 | .17 |
| Correlated 2-factor | 53 | 690.166 | 11124.15 | 1 | <.0001 | 13.02 | .98 | .039 |

Measurement invariance – we assessed measurement invariance for age and. Because of the large sample, we did not use chi-squared test statistics to evaluate model fit. Examining other fit indices, results indicated that there was measurement invariance between age categories at the level of factor configuration, factor loadings, item thresholds and item means. With respect to sex, results showed that there was measurement equivalence at all levels except the mean, indicating that in the NTR dataset, there were significant sex differences in the item means (Table S9).

**Table S9. Measurement invariance results**

|  | Model | df | Chi square | Fit indices | | | Deltas | |
| --- | --- | --- | --- | --- | --- | --- | --- | --- |
|  |  |  |  | **CSDT** | **CFI** | **RMSEA** | **ΔCFI** | **ΔRMSEA** |
| Age | Equal factor structure (configural invariance) | 159 | 502.873 | - | .980 | .033 | - | - |
|  | Factor loadings (metric invariance) | 179 | 471.717 | 0.053 | .983 | .029 | .003 | .004 |
|  | Thresholds (scalar invariance) | 191 | 496.951 | 0.014 | .981 | .030 | .001 | .003 |
|  | Means (means invariance) | 203 | 542.979 | <.001 | .979 | .030 | .002 | .000 |
| Sex | Equal factor structure (configural invariance) | 106 | 592.706 | - | .985 | .037 | - | - |
|  | Factor loadings (metric invariance) | 116 | 548.357 | <.001 | .986 | .034 | .001 | .003 |
|  | Thresholds (scalar invariance) | 132 | 601.776 | <.001 | .985 | .036 | .001 | .002 |
|  | Means (means invariance) | 144 | 1417.462 | **<.001** | .960 | .055 | **.025** | **.19** |

Note: CSDT – Chi square difference test. Please note that while some of the models showed a significant worsening of fit in the chi square difference test, it did not in the delta CFI and delta RMSEA tests. Because of the very large sample size of the NTR, for which the CSDT is very sensitive, we followed the latter fit indices difference tests (Meade et al. 2012).

## Conclusion

Reliability analyses in the three samples showed that the ASEBA derived scales were internally consistent. Exploratory factor analysis showed the presence of two factors (NA and SI), which was confirmed in the confirmatory factor analyses we performed in samples 2 and 3. When adding DS14 and ASEBA items to a two-factor model, this did not produce a worse fit compared to the 4-factor model in which DS14 and ASEBA factors were defined separately. Measurement invariance analysis with respect to age categories and sex showed that there was equality between age categories in the factor configuration, item loadings, and thresholds. In the general population, the item means differed for the subsequent age categories. For sex, similar findings were shown. Within the NTR dataset, there was measurement equivalence for age categories at all levels. With respect to sex, analysis concurred with the results of the general population sample, showing sex differences for item means. Construct validity analysis in the general population sample showed the ASEBA derived scales correlated in a similar degree and direction as DS14 derived scales to personality and mood constructs that were thought of as concordant (e.g., neuroticism and NA) and discordant (e.g., extraversion and SI). ROC analysis determined that with the cut-off of 3, the ASEBA derived NA and SI scales predicted the gold standard Type D classification very well, with good diagnostic accuracy. In sum, we conclude that the ASEBA derived proxy scales are valid and can be used reliably in the current and future research.

# References for Supplement

T.M. Achenbach, L.A. Rescorla. Manual for the ASEBA Adult Forms & Profiles. University of Vermont Research Center for Children, Youth, and Families, Burlington, VT (2003)

Denissen JJ, Geenen R, van Aken MA, Gosling SD, Potter J. Development and validation of a Dutch translation of the Big Five Inventory (BFI). J Pers Assess. 2008;90(2):152-7.

Denollet J (2005) DS14: Standard assessment of negative affectivity, social inhibition, and Type D personality. Psychosom Med 67(1):89-97.

Kroenke, Spitzer, & Williams. (2001). The PHQ-9. Journal of General Internal Medicine, 16(9), 606-613.

McDonald, R. P. (1999). Test theory: A unified treatment. L. Erlbaum Associates, Mahwah, N.J.

Meade AW, Johnson EC, Braddy PW. Power and sensitivity of alternative fit indices in tests of measurement

invariance. J Appl Psychol. 2008;93(3):568-92.

Putnick DL, Bornstein MH. Measurement Invariance Conventions and Reporting: The State of the Art and Future Directions for Psychological Research. *Dev Rev*. 2016;41:71‐90. doi:10.1016/j.dr.2016.06.004

Rhemtulla, M., Brosseau-Liard, P. É., & Savalei, V. (2012). When can categorical variables be treated as continuous? A comparison of robust continuous and categorical SEM estimation methods under suboptimal conditions. *Psychological methods*, *17*(3), 354.

Spitzer, Kroenke, Williams, & Löwe. (2006). A Brief Measure for Assessing Generalized Anxiety Disorder: The GAD-7. *JAMA Internal Medicine, 166*(10), 1092-1097.

Trevethan R. Sensitivity, Specificity, and Predictive Values: Foundations, Pliabilities, and Pitfalls in Research and Practice. Front Public Health. 2017;5:307. Published 2017 Nov 20. doi:10.3389/fpubh.2017.00

1. MI_model <- measEq.syntax(configural.model=HS.model, data = f2, ordered = c("ASEBA_67", "ASEBA_45", "ASEBA_42", "ASEBA_112", "ASEBA_111", "ASEBA_50", "ASEBA_75", "ASEBA_103", "ASEBA_69", "ASEBA_115", "ASEBA_48", "ASEBA_87"), ID.fac = "std.lv", parameterization = "delta" , ID.cat = "Wu.Estabrook.2016", group = "age_category", return.fit = TRUE)

   summary(MI_model, fit.measures=T)

   MI_model.loadings <- measEq.syntax(configural.model=HS.model, data = f2, ordered = c("ASEBA_67", "ASEBA_45", "ASEBA_42", "ASEBA_112", "ASEBA_111", "ASEBA_50", "ASEBA_75", "ASEBA_103", "ASEBA_69", "ASEBA_115", "ASEBA_48", "ASEBA_87"), ID.fac = "std.lv", parameterization = "delta" , ID.cat = "Wu.Estabrook.2016", group = "age_category", group.equal = ("loadings"), return.fit = TRUE)

   summary(MI_model.loadings, fit.measures=T) [↑](#footnote-ref-1)
