# Supplementary material for: The Heritability of Type D Personality by an Extended Twin-Pedigree Analysis in the Netherlands Twin Register
Source: Behav Genet. 2020 Oct 16;51(1):1–11. doi: 10.1007/s10519-020-10023-x (PMC7815549; doi:10.1007/s10519-020-10023-x)
Supplement: Supplementary file 2 — Supplementary file2 (DOCX 271 kb) [file 10519_2020_10023_MOESM2_ESM.docx]

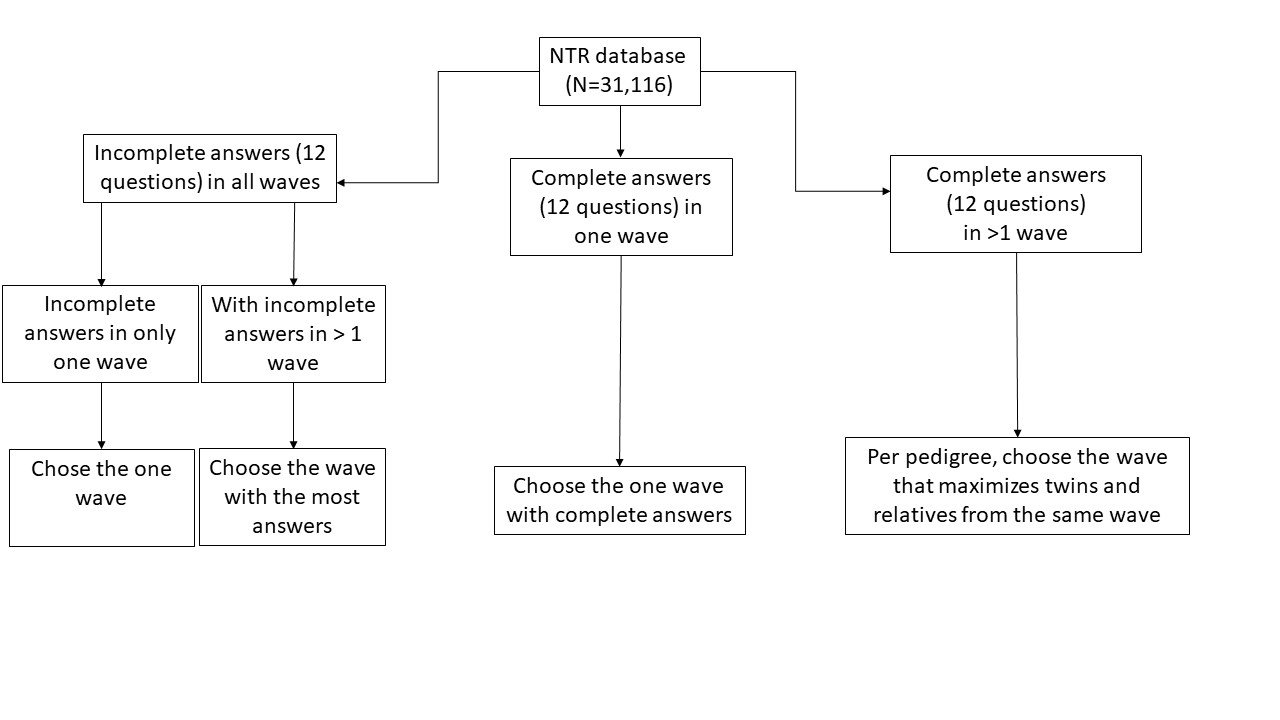


Supplemental Figure 1. The imputation scheme for the Type D personality proxy construction in the NTR.


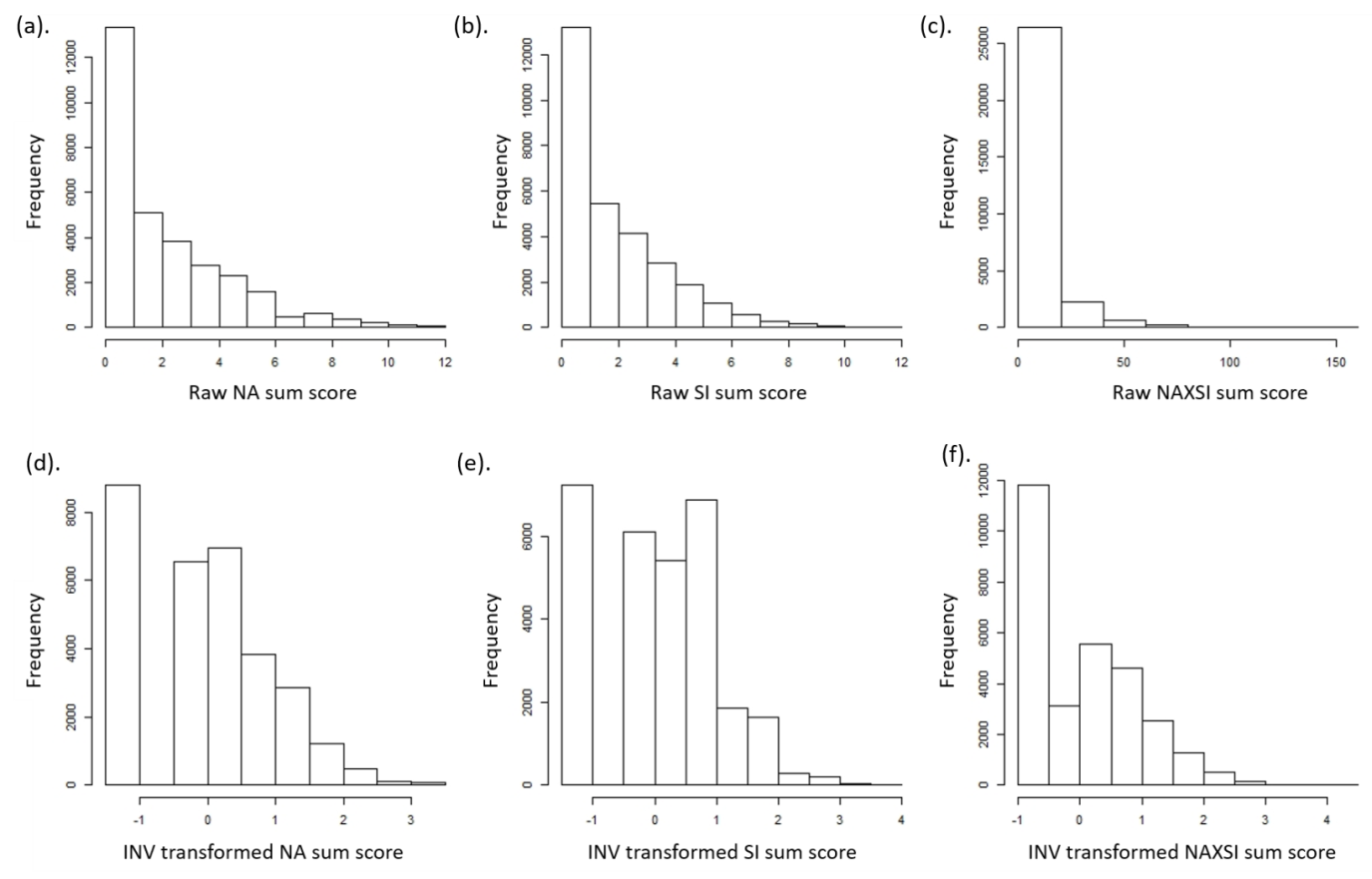


Supplemental Fig 2. NA, SI and NAxSI sum scores before and after inverse normal transformation

Note: (INV). (a-c) the distribution of NA, SI and NAxSI sum score on the raw scale; (d-e) the distribution of NA, SI and NAxSI sum score after INV transformation.

Supplemental Table 1. Parameter estimates (and SE) for variance components on the untransformed NA and SI scales

| Model | ∆df | Log-likelihood | χ^2^  (p-value) | Estimates of variance components  (standard error) | | | | | Broad-sense heritability  (standard error)** |
| --- | --- | --- | --- | --- | --- | --- | --- | --- | --- |
|  |  |  |  | A | D | H | E | Total variance |  |
| NA | | | | | | | | | |
| ADE plus* household | -- | - 40853.27 | -- | 0.68 (0.097) | 1.91 (0.086) | 0.48 (0.078) | 2.30 (0.10) | 5.37 (0.18) | 0.48 (0.011) |
| ADE no household | 1 | - 40873.64 | 40.74 (<.001) | 0.79 (0.092) | 1.90 (0.087) | -- | 2.67 (0.083) | 5.36 (0.15) | 0.50 (0.011) |
| AE plus household | 1 | - 41069.27 | 391.26 (<.001) | 1.67 (0.10) | -- | 0.46 (0.078) | 3.26 (0.11) | 5.39 (0.17) | 0.31 (0.014) |
| SI | | | | | | | | | |
| ADE plus* household | -- | - 35056.67 | -- | 0.45 (0.071) | 1.57 (0.066) | 0.31 (0.052) | 1.71 (0.074) | 4.04 (0.13) | 0.50 (0.011) |
| ADE no household | 1 | - 35075.82 | 38.3 (<.001) | 0.53 (0.068) | 1.57 (0.066) | -- | 1.93 (0.064) | 4.04 (0.11) | 0.52 (0.011) |
| AE plus household | 1 | - 35293.28 | 473.22 (<.001) | 1.11 (0.077) | -- | 0.32 (0.052) | 2.62 (0.084) | 4.04 (0.13) | 0.27 (0.015) |
| NAXSI | | | | | | | | | |
| ADE plus*  household | -- | - 89554.08 | -- | 12.80 (3.16) | 56.40 (2.69) | 16.85 (2.89) | 76.91 (3.58) | 162.96 (6.20) | 0.42 (0.013) |
| ADE no household | 1 | - 89572.73 | 37.3 (<.001) | 15.73 (3.01) | 56.93 (2.70) | -- | 90.02 (2.83) | 162.67 (4.94) | 0.45 (0.012) |
| AE plus household | 1 | - 89744.86 | 381.56 (<.001) | 41.65 (3.35) | -- | 18.89 (2.96) | 103.16 (4.03) | 163.70 (6.02) | 0.25 (0.016) |

∆df: the difference of degree of freedom; χ2 (p-value): likelihood ratio test statistic calculated by -2 (log likelihood sub-nested model – log likelihood full model); p-value: log-likelihood ratio test p-values; A: additive genetic variance; D: dominance genetic variance; H: shared household variance; E: non-genetic variance.

* The final selected model based on ∆χ^2^

** Broad-sense heritability column gives the proportion of variance explained by additive and dominance genetic variance.

Supplemental Table 2. The estimations of variance, covariance and correlations derived from the best fitting bivariate genetic model for negative affectivity and social inhibition.

| component | SI-SI variance (standard error) | NA-NA variance (standard error) | SI-NA covariance (standard error) | Total SI-NA covariance | % phenotypic covariance of NA-SI  (standard error) | Correlation (r) |
| --- | --- | --- | --- | --- | --- | --- |
| A | 0.094 (0.015) | 0.10 (0.014) | 0.057 (0.011) | 0.42 (0.020) | 13.6% (2.3%) | 0.57 |
| D | 0.32 (0.014) | 0.28 (0.013) | 0.21 (0.010) |  | 50.0% (2.4%) | 0.69 |
| H | 0.067 (0.011) | 0.067 (0.010) | 0.063 (0.0083) |  | 15.0% (1.8%) | 0.94 |
| E | 0.36 (0.015) | 0.34 (0.015) | 0.090 (0.011) |  | 21.4% (2.2%) | 0.26 |

A: additive genetic component, D: dominance genetic component, H: share household effects, E: unique environmental effects, SI: social inhibition, NA: negative affectivity.
